# Data on the bipolar electroactive conducting polymers for wireless cell stimulation

**DOI:** 10.1016/j.dib.2020.106406

**Published:** 2020-10-11

**Authors:** Chunyan Qin, Zhilian Yue, Yunfeng Chao, Robert J. Forster, Fionn Ó Maolmhuaidh, Xu-Feng Huang, Stephen Beirne, Gordon G. Wallace, Jun Chen

**Affiliations:** a ARC Centre of Excellence for Electromaterials Science, Intelligent Polymer Research Institute, Australian Institute for Innovative Materials, Innovation Campus, University of Wollongong, Squires Way, North Wollongong, NSW 2519, Australia; b National Centre for Sensor Research, School of Chemical Sciences, Dublin City University, Dublin 9, Ireland; c Illawarra Health and Medical Research Institute, School of Medicine, University of Wollongong, Wollongong, NSW 2522, Australia

**Keywords:** Cell stimulation, Bipolar electroactive, Wireless, Conducting polymer, Bipolar electrostimulation, 3D printing, PPy

## Abstract

Data in this article is associated with our research article “Bipolar Electroactive Conducting Polymers for Wireless Cell Stimulation” [1]. Primarily, the present article shows the data of PPy-*p*TS, PPy-DS and PPy-DS/collagen in conventional electrochemical process and bipolar electrochemical process for comprehensive supplement and comparison to help with better understanding and developing conducting polymers based bipolar electrochemistry. Secondly, the presented data of bipolar electrostimulation (BPES) protocol development constitute the complete dataset useful for modeling the bipolar electroactive conducting polymers focusing on wireless cell stimulation, which are reported in the main article. All data reported were analysed using Origin 2018b 64Bit.

## Specifications Table

**Table:** 

Subject	Chemistry
Specific subject area	Bipolar Electrochemistry for Wireless Cell Stimulation
Type of data	Graph Figure Table
How data were acquired	CHI-720 Electrochemical Analyzer system Shimadzu UV–vis 3600 HR800 Raman spectrometer IRpretige-21, Shimadzu Scanning electron microscopy (JEOL JSM-7500FA) ZEISS Axiovert microscope (Carl Zeiss, Germany)
Data format	Raw Analyzed
Parameters for data collection	All experiments for conducting polymers characterizations were carried out in PBS (pH=7.4) environment. PC 12 cells were seeded and cultured in DMEM growth media supplemented with 2 mM glutamine, 5% (v/v) FBS and 10% (v/v) horse serum in a humidified 37 °C incubator with 5% CO_2_ atmosphere before further use. For differentiation, a low serum medium (1% horse serum) supplemented with 50 ng/ml NGF was used.
Description of data collection	Primary data (i.e. raw materials, operating data) were collected via operational software with the instruments/manufacturers introductions. Data was processed using Origin 2018b 64Bit, for purposes of data analysis and diagram presentation.
Data source location	Institute: Intelligent Polymer Research Institute/University of Wollongong City/Town/Region: North Wollongong Country: Australia
Data accessibility	With the article and in Mendeley, http://dx.doi.org/10.17632/4zgkyfxhcd.1
Related research article	C. Qin, Z. Yue, Y. Chao, R.J. Forster, F.Ó. Maolmhuaidh, X.F. Huang, S. Beirne, J. Chen, G.G. Wallace, Bipolar electroactive conducting polymers for wireless cell stimulation, https://doi.org/10.1016/j.apmt.2020.100804.

## Value of the Data

• The presented data constitute the complete dataset useful for modeling the electroactive doped conducting polymers focusing on the bipolar electrochemistry in wireless cell stimulation point of view.
• The data could be used by researchers as starting point when studying or developing similar conducting polymers based bipolar electrochemistry.
• The data can support similar analysis, considering both conventional three-electrode system and bipolar electrochemical system in biological environment (PBS buffer) first, further insights could consider about applications in wireless cell stimulation as we proved.
• All findings have been adopted to support conducting polymers based bipolar electrochemistry involved in studies related to wireless cell stimulation, which advances the field of biomedical stimulation and controlled release system.

## 1 Data Description

**Fig. 1:**
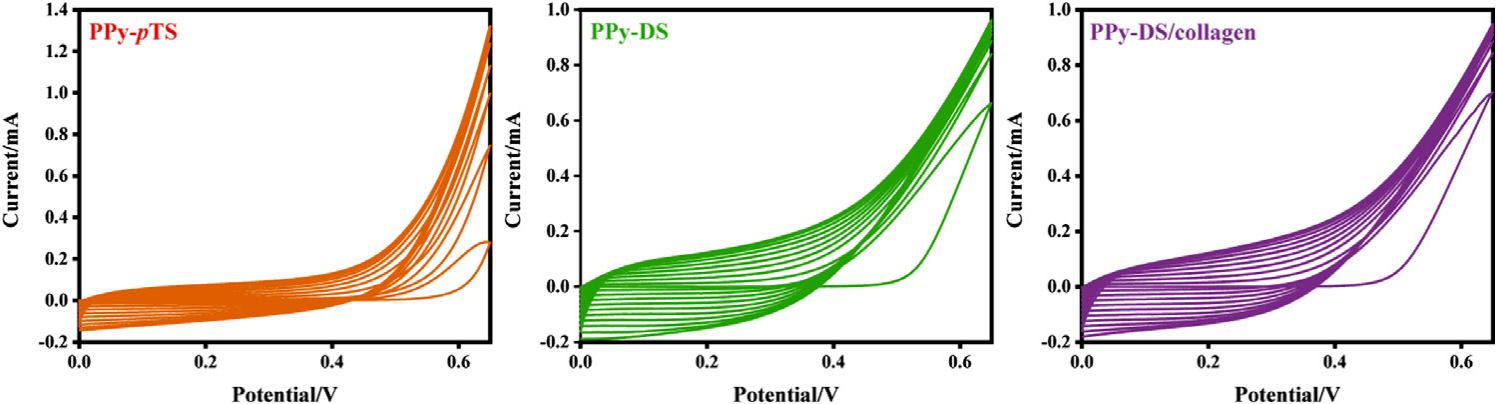
**Electrodeposition of polyporrole (PPy) films.** Cyclic voltammograms of PPy-*p*TS, PPy-DS and PPy-DS/collagen films grown onto FTO glass obtained during their synthetic process. Aqueous solution contained 0.2 M Py with 0.1 M *p*TS, 2 mg/ml DS without or with 2 µg/ml collagen respectively. Potentials were recorded vs. a Ag/AgCl reference electrode under scan range of 0–0.65 V at a scan rate of 20 mV/s (10 cycles).

**Fig. 2:**
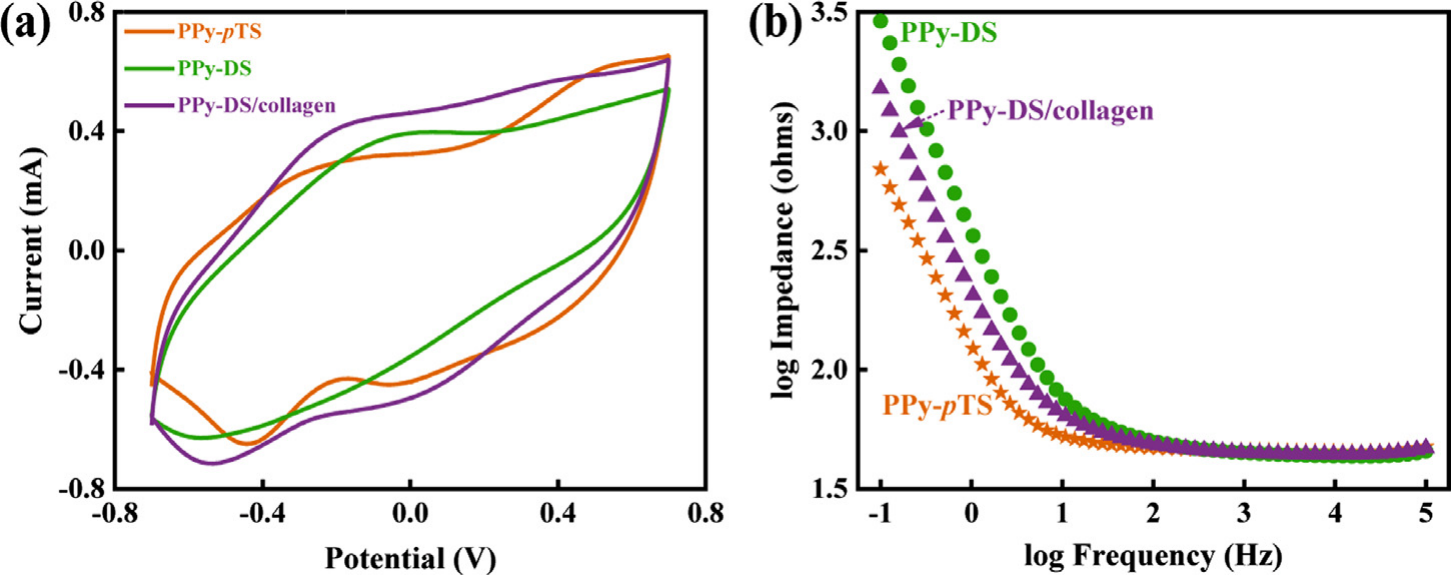
**Electrochemical activity of PPy films in PBS (pH = 7.4).** (a) Cyclic voltammetry (CV) of PPy-DS/collagen, PPy-DS and PPy-*p*TS at a scan rate of 100 mV/s. (b) electrochemical impedance spectroscopy (EIS) of them over a frequency range of 0.1–10^5^ Hz.

**Fig. 3:**
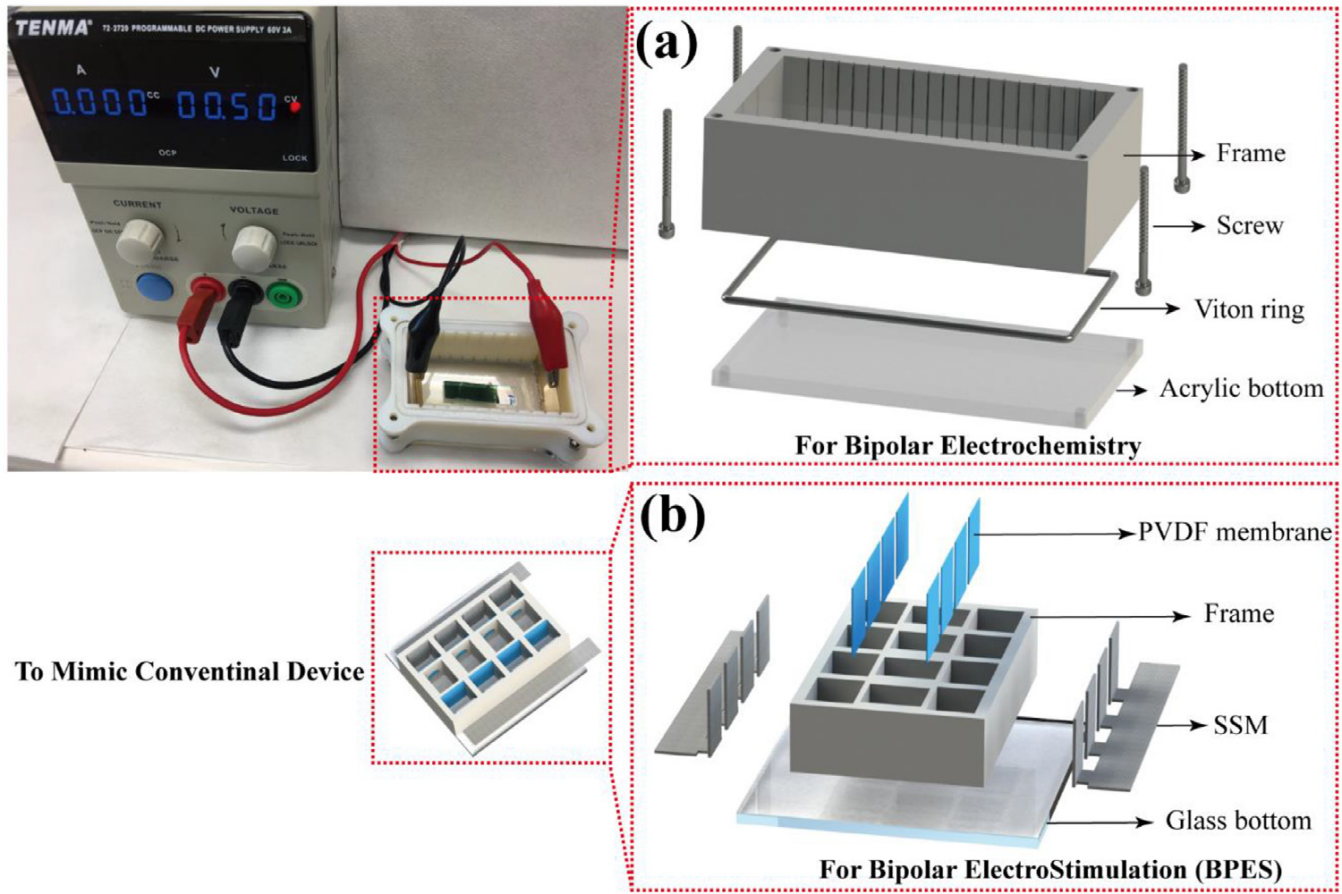
**Schematic diagram of the cells for (a) bipolar electrochemistry and devices for (b) bipolar electrostimulation (BPES).** In (a), viton ring and the four screws were used to fix the frame closely onto the acrylic bottom. For more convenient observation and application in following in situ spectro-electrochemical experiments, clear acrylic bottom replaced the opaque one. In (b), all parts were carefully positioned and fixed on the glass bottom using silicon adhesive sealant. The SSM strips extending beyond the frame were designed to connect with the external DC power supply. The cell culture wells (1.0 cm^2^/well) were in the middle of the device, which mimicked the one used in conventional electrochemical cell stimulation system.

**Fig. 4:**
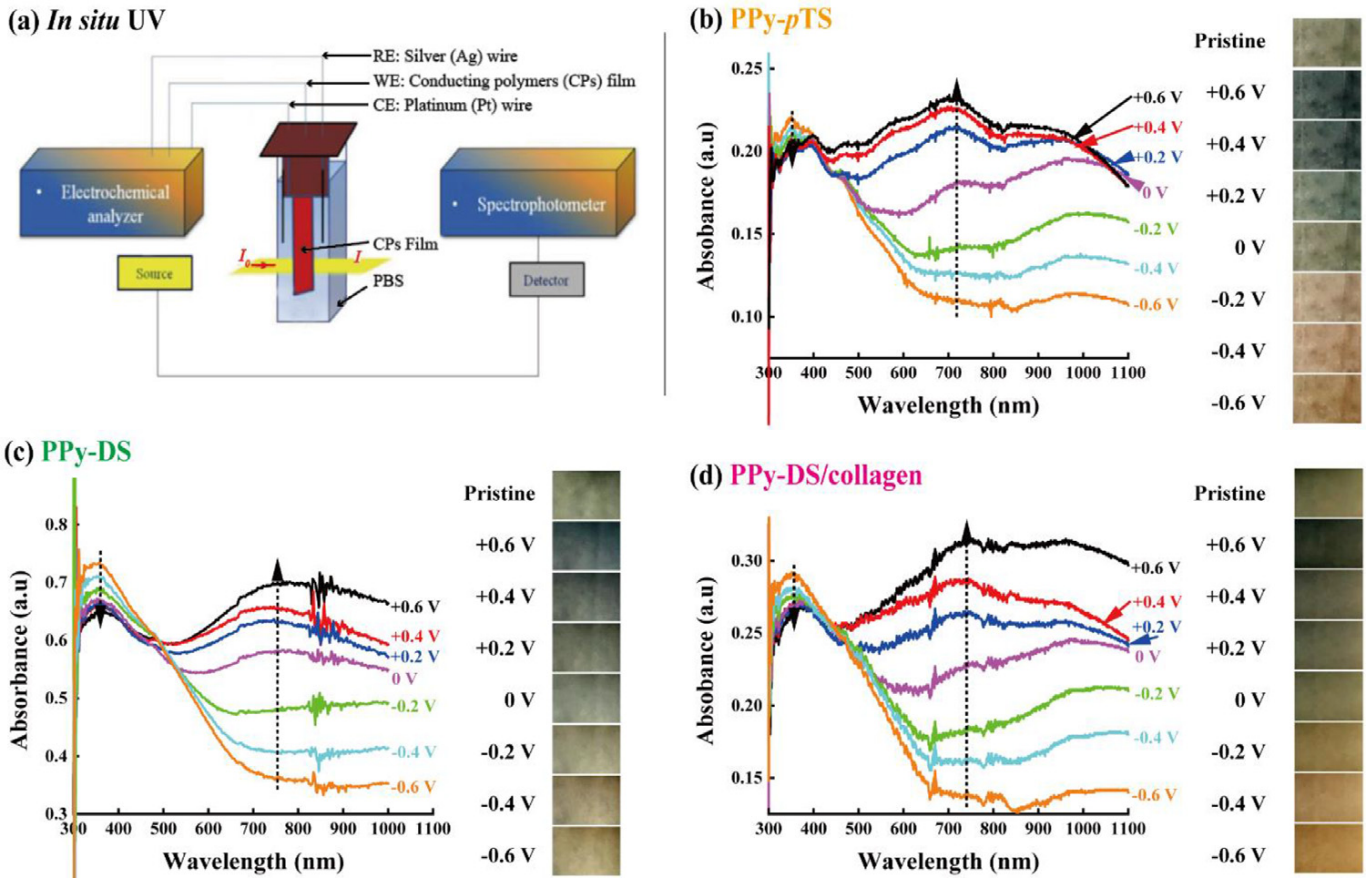
***In situ* UV–vis absorption spectra combined with the conventional three-electrode electrochemical system.** (a) Schematic illustration of spectro-electrochemical configuration. A custom-made quartz cuvette acting as an electrochemical reaction cell was equipped with a CP film (1 cm × 0.8 cm) as a working electrode, silver (Ag) wire as a reference electrode and platinum (Pt) wire as a counter electrode. *In situ* UV–visible absorption spectra in the range of 300–1100 nm and according colours of (b) PPy-*p*TS, (c) PPy-DS and (d) PPy-DS/collagen obtained after applied consecutive potentials from −0.6 V to +0.6 V. All potentials are vs. a Ag wire reference electrode and keep 30 s poise time in PBS (pH = 7.4).

**Fig. 5:**
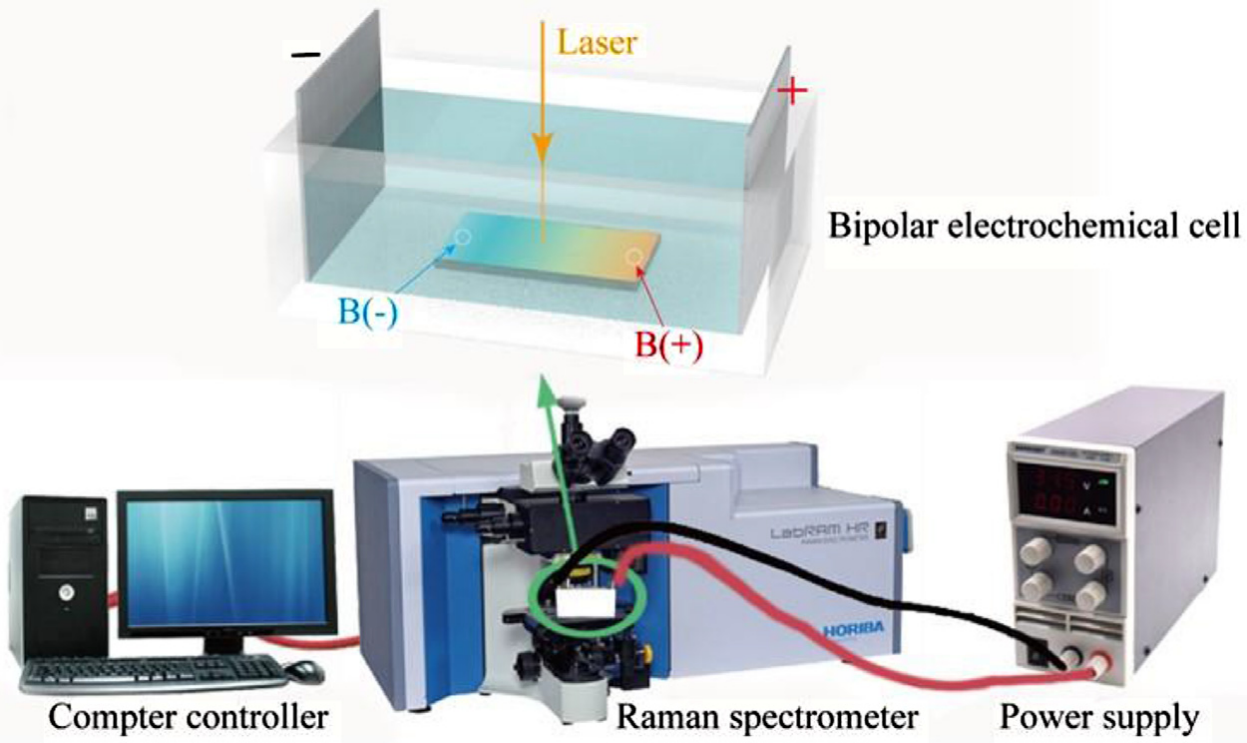
**Schematic illustration of in situ Raman measurement along with bipolar electrochemistry process.** A micro-Raman spectrometer with 632.81 nm diode laser excitation was utilized. The objective len (× 50 WLD) was positioned directly above the optical window and focused on the spot of bipolar electrode. The laser spot was around 1 - 2 micrometer in diameter with a lower laser power (less than 10 mW) to avoid possible laser irradiation damage of samples. All spectra were acquired by 10 s data collection within the wavenumber range of 500–2000 cm^−1^. All samples were placed in the middle of designed bipolar cell, immersed into the PBS. For reference, the pristine spectrum was taken from the original film in PBS without the applied driving voltage, and the recovered spectrum showed the Raman spectrum from the film after bipolar testing in PBS with removed driving voltage 1 min later. All the in situ spectra were obtained with different driving voltage for 30 s. B(-) and B(+) present the Raman spectra from the poles of bipolar electrode, which were opposite the cathodic and anodic driving electrodes. According to the principle of bipolar electrochemistry, the oxidation at B(-) site and reduction at B(+) site occurred at the same time.

**Fig. 6:**
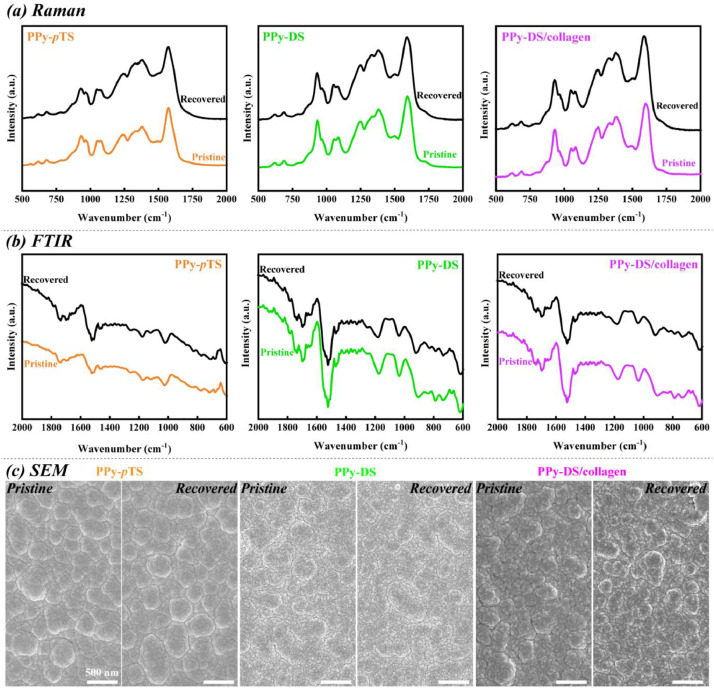
**Characterizations of synthesized PPy-*p*TS, PPy-DS and PPy-DS/collagen before (pristine) and after (recovered) bipolar electrochemical testing.** (a) Raman spectrometry, (b) FTIR spectrometry and (c) scanning electron microscopy (SEM) were performed under identical bipolar testing condition. All samples were tested in PBS under 5.5 V driving voltage for 30 s, and then taken out from PBS, subsequently rinsed with Milli-Q water and allowed to dry before all *ex situ* characterizations.

**Fig. 7:**
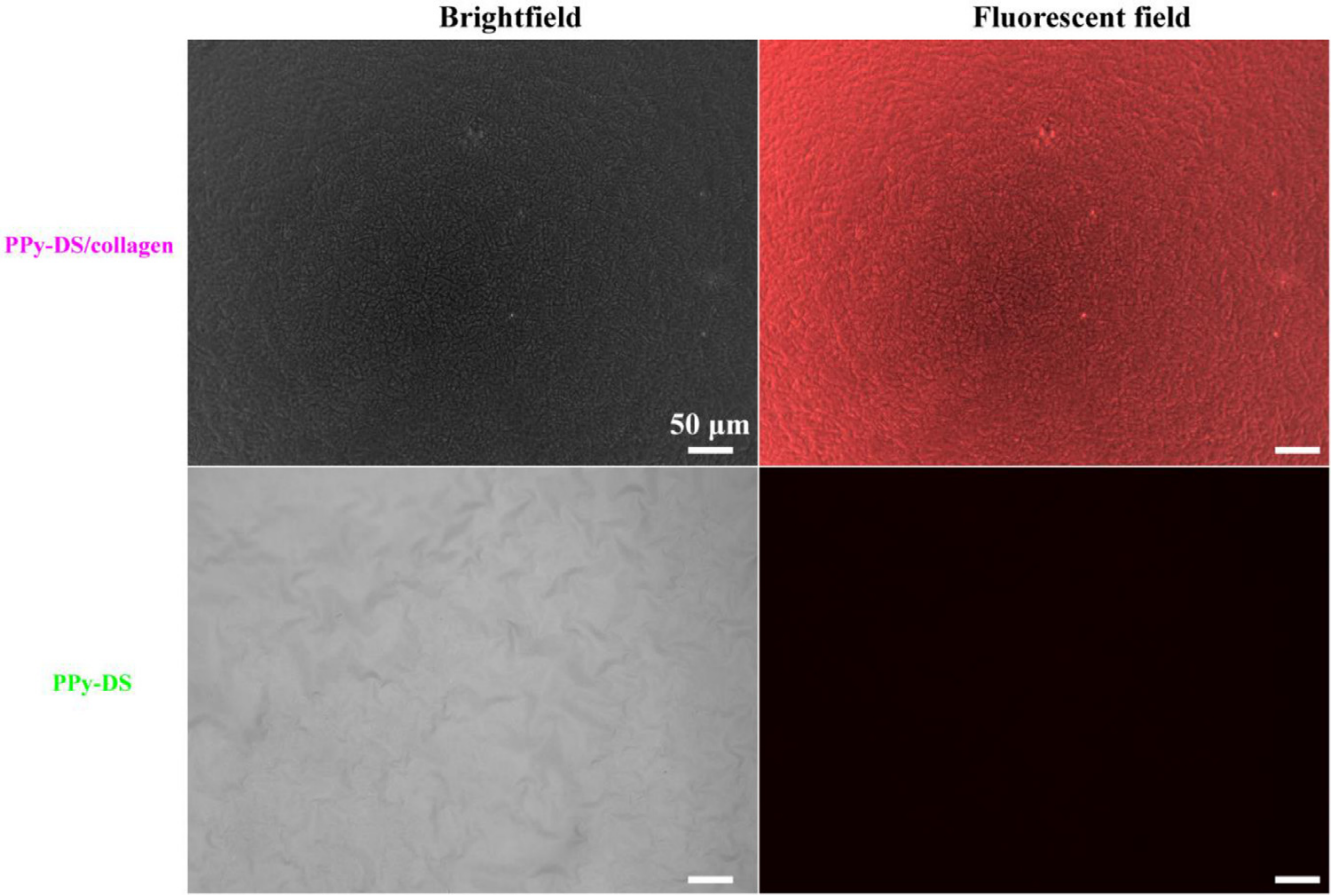
**Images of PPy-DS and PPy-DS/collagen after fluorescent labeling.** PPy-DS and PPy-DS/collagen (1 cm × 1 cm) were firstly soaked in 1.5 ml solution with isometric PBS and ethanol for 30 min. Then 500 µl rhodamine red™-X succunimidyl ester/DMSO at a final concentration of 2.5 µg/ml was added into each well, followed by 5 h’ reaction at RT in covered Al foil as darkroom. Finally, replaced with the fresh PBS after thorough washing with isometric PBS and ethanol for 10 min each time, total three times to remove the residue fluorescent dye. The surface morphology of fluorescently labelled samples were examined in using a ZEISS Axiovert microscope.

**Fig. 8:**
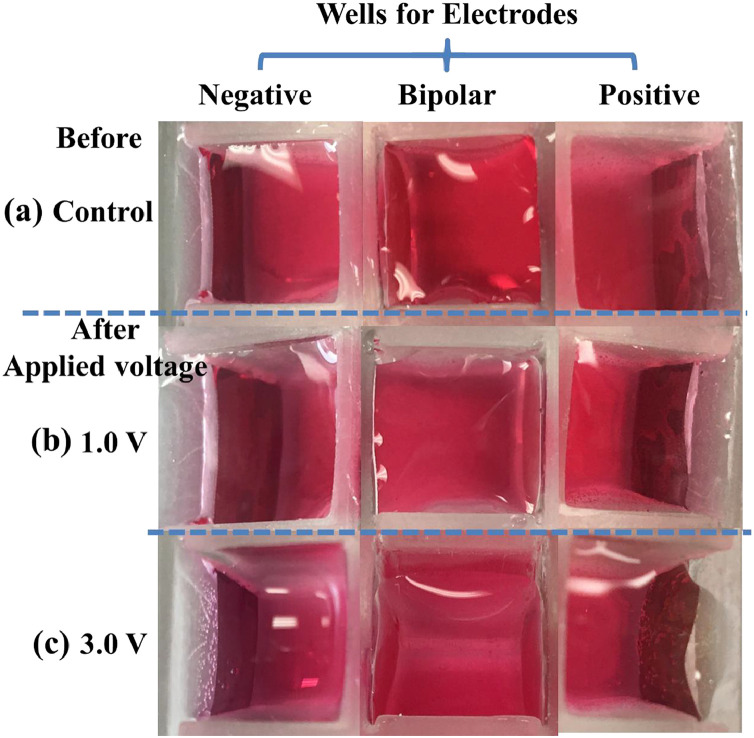
Images acquired from different wells for negative feeder electrode, bipolar electrode, and positive feeder electrode, respectively, before (control: (a)) and after applied (b) 1.0 V, (c) 3.0 V.

**Fig. 9:**
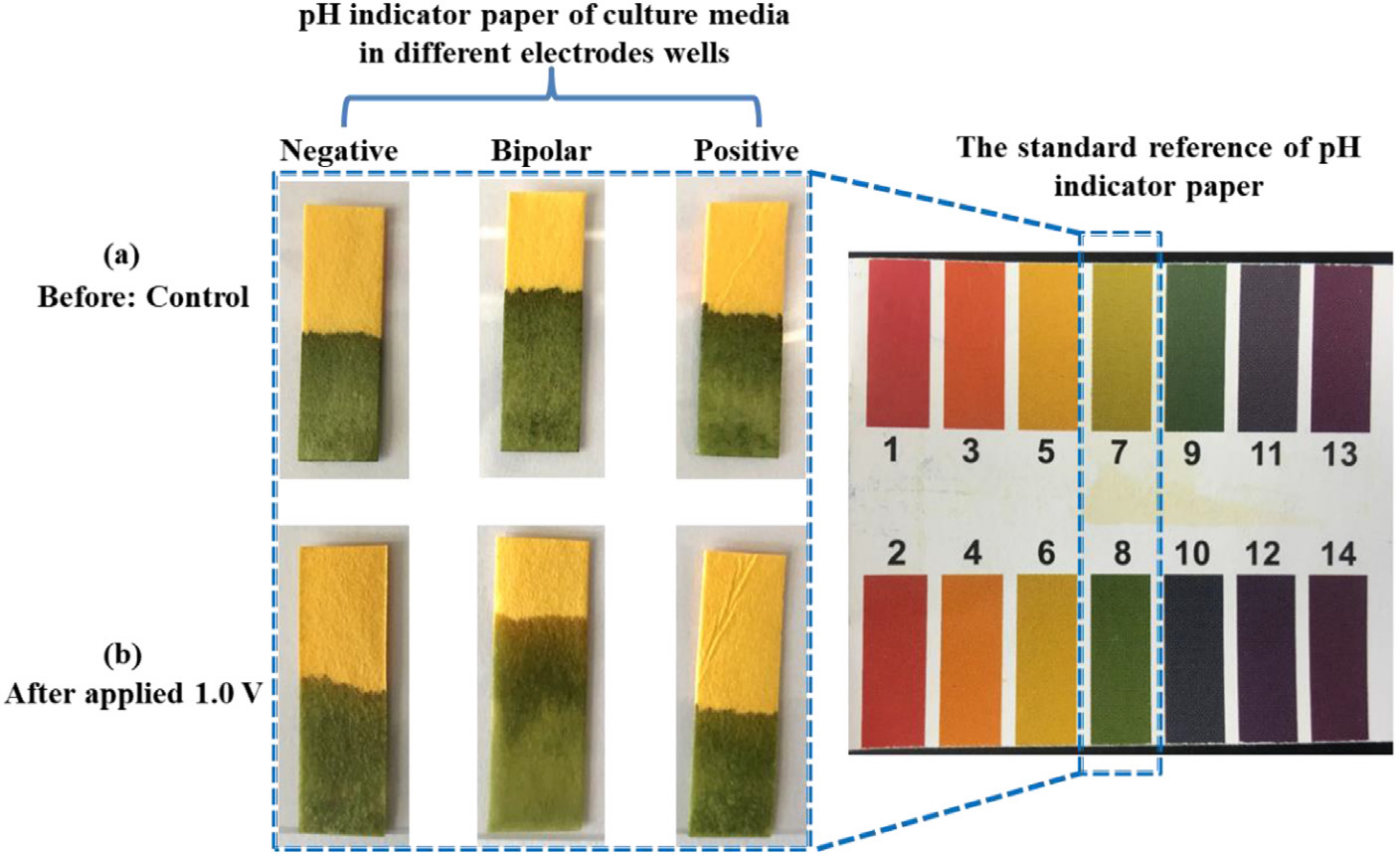
Images of pH indicator papers acquired from culture media in different wells for negative feeder electrode, bipolar electrode, and positive feeder electrode respectively, before ((a) control) and after (b) applied 1.0 V underwent identical bipolar electrostimulation process.

**Fig. 10:**
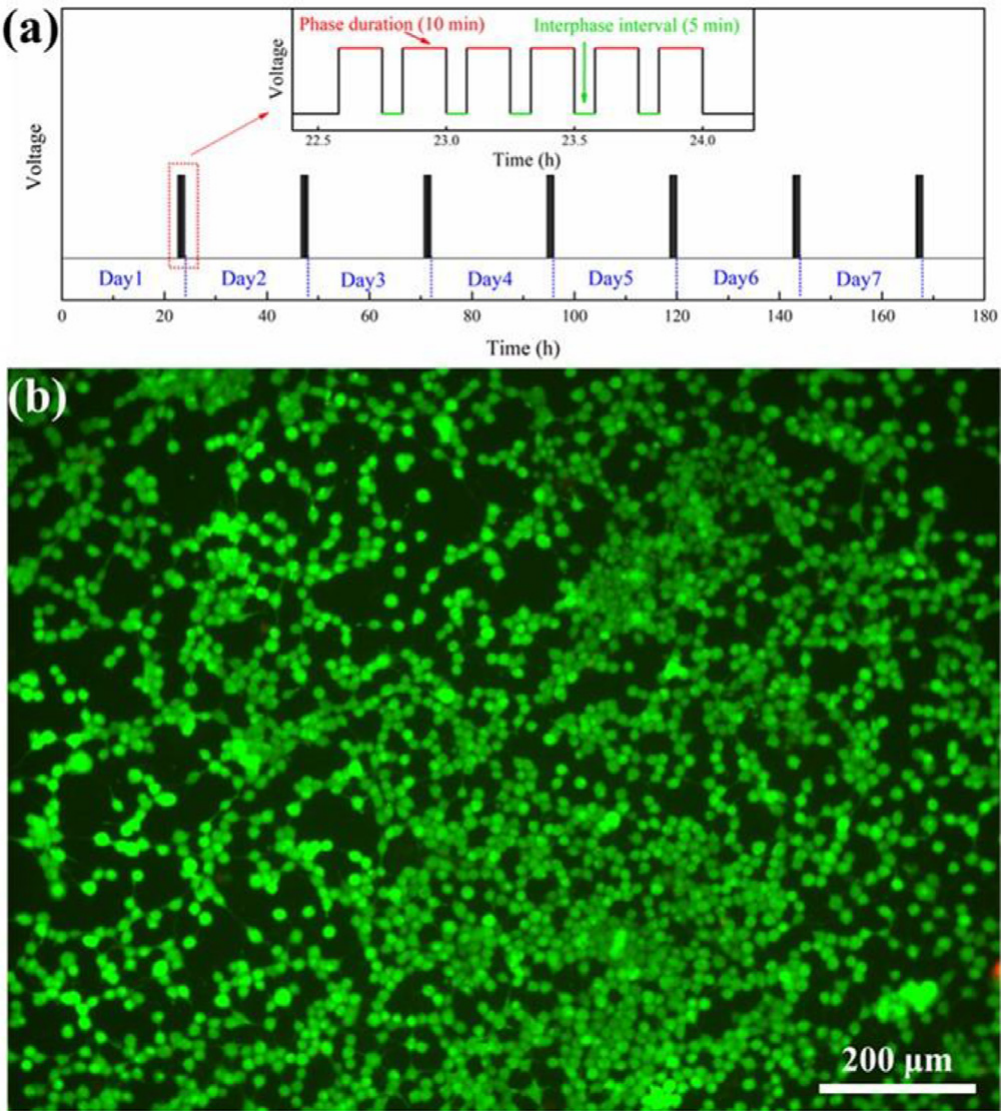
**Cell viability under pulse mode of BPES.** (a) pulse mode consists phase duration of 10 min with interphase interval of 5 min (waveform as inserted) to form total 1 h per day in consecutive 7 days. (b) Image of PC 12 cells on day 7 via live (calcein AM; green) and dead (PI; red) staining, after cultured on PPy-DS/collagen with pulse mode of BPES (1.0 V DC driving voltage) in growth media.

**Fig. 11:**
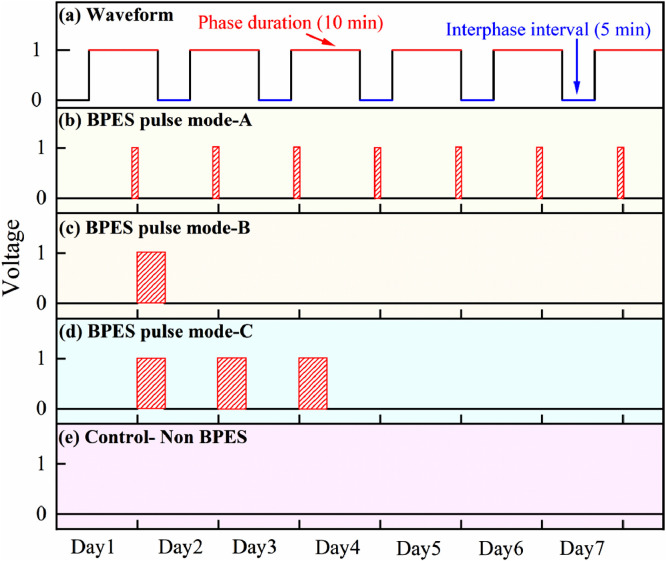
**Schematic of programmed BPES pulse modes.** (a) The waveform of the applied pulse mode comprised phase duration of 10 min with interphase interval of 5 min. (b) BPES pulse mode-A: cells were stimulated for 1 h per day over 7 days. (c) BPES pulse mode-B: cells were stimulated for 8 h on day 2, then cultured as normal in the absence of BPSE for the following 5 days. (d) BPES pulse mode-C: cells were stimulated on day 2 for 8 h, and this pattern was continued until day 5, after which they were cultured as normal in the absence of BPSE to the end of day 7. (e) Control Non-BPES: cells were cultured under identical conditions without BPES (i.e. normal condition). All experiments were conducted over a period of 7 days and cells fixed on day 7 for immunofluorescence staining.

**Fig. 12:**
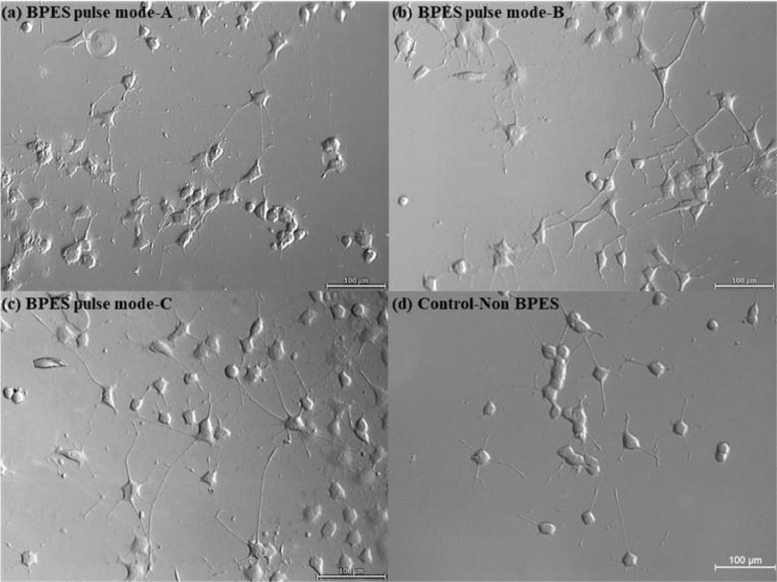
Phase-contrast images of PC 12 cells on day 7 after experiencing different BPES pulse modes.

The data presented in this article are related to the research article, “Bipolar Electroactive Conducting Polymers for Wireless Cell Stimulation” [1]. The cyclic voltammograms data of synthesizing the PPy-*p*TS, PPy-DS and PPy-DS/collagen materials are presented in Fig. 1. The dopants on polypyrrole (PPy) matrix vary from typically small size dopant, *p*-toluenesulfonic acid monohydrate (*p*TS) to bioinert high molecular weight dopant, dextran sulfate sodium salt from Leuconostoc spp. (DS) and collagen (Type I from rat tail). Comparative CVs and electrochemical impedance spectroscopy (EIS) of these three doped PPy films in PBS are shown in Fig. 2 [2]. Fig. 3 shows the schematic diagram of the devices for bipolar electrochemistry and bipolar electrostimulation (BPES). All spectro-electrochemical data (in situ UV–vis with conventional electrochemical system in Fig. 4, in situ Raman spectrometry with bipolar electrochemical system in Fig. 5, *ex situ* Raman spectrometry and FTIR spectrometry data before and after underwent bipolar electrochemical process in Fig. 6) were obtained and analysed to identify the bipolar electrochemical activation was related to the typical electrochemical redox process within PPy films induced by electric field switching [3]. Fig. 7 displays the images of PPy-DS and PPy-DS/collagen after fluorescence labeling using a ZEISS Axiovert microscope. The BPES protocol for the specific-designed device was developed by optimizing the applied DC voltage under pulse mode. Images of culture media in Fig. 8, pH indicator papers in Fig. 9, cell viability in Fig. 10 and schematic illustrations of the waveform of applied pulse mode and three distinct programmed BPES pulse modes used for studies of cell proliferation and cell differentiation in Fig. 11 are reported. The corresponding data in Fig. 12 and Table 1 are comprehensive supplement for data analysis of BPES on cell differentiation. One compressed file that contains all raw data of the graphs being shown (Electrodeposition data in Fig. 1, CV and EIS data in Fig. 2, In situ UV data in Fig. 4, Ex situ FTIR and Raman data in Fig. 6) can be available in Mendeley Data, DOI: 10.17632/4zgkyfxhcd.1.

**Table 1:** Results of different BPES pulse modes on cell differentiation.

Modes	Neurites number	Neurite length (µm)		
		Total	Mean	Max
Control	4.3 ± 0.4	231.9 ± 18.9	52.9 ± 11.7	81.5 ± 12.6
BPES pulse mode-A	8.5 ± 1.0	761.6 ± 166.8	84.6 ± 27.5	182.8 ± 29.3
BPES pulse mode-B	6.8 ± 1.5	529.3 ± 117.5	77.9 ± 3.9	95.6 ± 2.2
BPES pulse mode-C	9.2 ± 1.3	1524.9 ± 191.3	166.5 ± 14.4	203.6 ± 17.7

Data are represented as mean ± standard deviation (SD).

## 2 Experimental Design, Materials and Methods

### 2.1 Materials synthesis

The preparation of polypyrrole (PPy) films were carried out by cyclic voltammetry (CV) using an CHI-720 Electrochemical Analyzer system in a standard three-electrode electrochemical cell, which configures a platinum (Pt) sheet counter electrode (1 cm × 3 cm), an Ag/AgCl reference electrode, and a FTO-glass working electrode (1 cm × 2 cm). Depositions were obtained from an aqueous solution containing 0.2 M distilled pyrrole (Py) with 0.1 M *p*TS, 2 mg/ml DS without or with 2 µg/ml collagen within a potential range of 0–0.65 V at a scan rate of 20 mV/s. Different dopants were employed to obtain various PPy*-p*TS, PPy-DS and PPy-DS/collagen films in order to investigate the effects of dopants on bipolar electrochemical activities. After polymerization, all films were thoroughly rinsed with Milli-Q water and allowed to dry under ambient conditions before further characterization and investigation.

### 2.2 Design and methods

CVs of synthesized PPy-*p*TS, PPy-DS and PPy-DS/collagen were carried out with a potential range of −0.7 V to +0.7 V at a scan rate of 100 mV/s, while EIS were performed over the frequency range of 0.1 Hz to 100 kHz using an AC signal with +50.0 mV vs the reference electrode, using the CHI-720 Electrochemical Analyzer system. *In situ* UV–vis spectra (Shimadzu UV–vis 3600) were recorded simultaneously with the conventional three-electrodes electrochemical system within the range of 300–1100 nm under different applied potentials (from −0.6 V to +0.6 V) in PBS (pH = 7.4). *In situ* and *Ex situ* Raman spectra (HR800 Raman spectrometer, Japan) were obtained by 10 s data collection within the wavenumber range of 500 cm^−1^ - 2000 cm^−1^, using excitation laser at 632.81 nm with a low laser power (less than 10 mW) and × 50 WLD objective lens. FTIR spectra were collected using a FT-IR spectrometer (IRpretige-21, Shimadzu) over a range of 600 cm^−1^ - 2000 cm^−1^. The surface morphology of fluorescently labelled PPy-DS and PPy-DS/collagen samples were examined in using a ZEISS Axiovert microscope.

## Declaration of Competing Interest

The authors declare that they have no known competing financial interests or personal relationships which have, or could be perceived to have, influenced the work reported in this article.
